# Resistance to Azoles in *Candida parapsilosis* Isolates from Spain Is Associated with an Impairment in Filamentation and Biofilm Formation

**DOI:** 10.3390/jof11040299

**Published:** 2025-04-09

**Authors:** Alba Torres-Cano, Cristina de Armentia, Alejandra Roldán, Elena López-Peralta, Juliana Manosalva, Paloma Merino-Amador, Fernando González-Romo, Mireia Puig-Asensio, Carmen Ardanuy, María Teresa Martín-Gómez, Daniel Romero-Herrero, Ana Pérez-Ayala, Marta López-Lomba, María Teresa Durán-Valle, Isabel Sánchez-Romero, María Muñoz-Algarra, María Pía Roiz-Mesones, Isabel Lara-Plaza, Maite Ruíz Pérez de Pipaón, Gregoria Megías-Lobón, María Ángeles Mantecón-Vallejo, Laura Alcázar-Fuoli, Diego Megías, Oscar Zaragoza

**Affiliations:** 1 Mycology Reference Laboratory, National Centre for Microbiology, Instituto de Salud Carlos III, Carretera Majadahonda-Pozuelo, Km2, 28220 Majadahonda, Madrid, Spain; atorres@isciii.es (A.T.-C.);; 2 Advanced Optical Microscopy Unit, Central Core Units, Instituto de Salud Carlos III, 28220 Majadahonda, Madrid, Spain; 3 Microbiology Department, University Hospital Clínico San Carlos, 28040 Madrid, Spain; 4 Fundación para la Investigación Biomédica del Hospital Clínico San Carlos (IdISSC), Department of Medicine, Complutense University, School of Medicine, 28040 Madrid, Spain; 5 Department of Infectious Diseases, Bellvitge University Hospital, 08097 Barcelona, Cataluña, Spain; 6 Bellvitge Biomedical Research Institute (IDIBELL), 08907 Barcelona, Cataluña, Spain; 7 Biomedical Research Networking Centre in Infectious Diseases (CIBERINFEC, CB21/13/00009), Instituto de Salud Carlos III, 28029 Madrid, Spain; 8 Microbiology Department, Bellvitge University Hospital, 08907 Barcelona, Cataluña, Spain; 9 Biomedical Research Networking Centre in Infectious Diseases in Respiratory Diseases (CIBERES CB06/06/0037), Instituto de Salud Carlos III, 28029 Madrid, Spain; 10 Department of Microbiology, Vall d’Hebron University Hospital, Universitat Autònoma de Barcelona, 08035 Barcelona, Cataluña, Spain; 11 Microbiology Unit, University Hospital 12 de Octubre, 28041 Madrid, Spain; 12 Research Institute from Hospital 12 de Octubre i + 12, 28041 Madrid, Spain; 13 Microbiology and Parasitology Department, Móstoles University Hospital, 28935 Madrid, Spain; 14 Microbiology Department, Puerta de Hierro University Hospital, 28222 Majadahonda, Madrid, Spain; 15 Microbiology Department, Marqués de Valdecilla University Hospital, 39008 Santander, Cantabria, Spain; 16 Valdecilla Research Instituto (Instituto de Investigación Valdecilla, IDIVAL), 39008 Santander, Cantabria, Spain; 17 Biomedical Research Networking Centre in Infectious Diseases CIBERINFEC (CB21/13/00068), Instituto de Salud Carlos III, 28029 Madrid, Spain; 18 Clinical Unit of Infectious Diseases, Microbiology and Parasitology, Virgen del Rocío University Hospital, 41013 Seville, Andalucía, Spain; 19 Biomedical Research Networking Centre in Infectious Diseases CIBERINFEC (CB21/13/00006), Instituto de Salud Carlos III, 28029 Madrid, Spain; 20 Clinical and Molecular Microbiology Group, Instituto de Biomedicina de Sevilla, HUVR/CSIC/Sevilla University, 41013 Seville, Andalucía, Spain; 21 Department of Clinical Microbiology, Burgos University Hospital, 09006 Burgos, Castilla y León, Spain; 22 Biomedical Research Networking Centre in Infectious Diseases CIBERINFEC (CB21/13/00105), 28029 Madrid, Spain

**Keywords:** *Candida parapsilosis*, azole resistance, pseudohyphae, biofilm, adhesion, antifungal, disinfectant

## Abstract

In recent years, there has been an increase in the incidence of fluconazole-non-susceptible (FNS) *Candida parapsilosis*. The reasons why these strains are able to colonize hospitals remain unknown. It is also unclear whether these strains exhibit resistance to the disinfectants used in hospitals, facilitating their spread. For these reasons, in this work, we aimed to investigate whether fluconazole resistance was associated with virulence traits and the resistance of these strains to common hospital disinfectants. The general conclusion of the study was that more than 95% of the FNS strains, regardless of the resistance mutation they carried, had filamentation problems, whereas around 75% of the susceptible strains formed pseudohyphae and were capable of filamentation. This 95% of the FNS strains did not form pseudohyphae, did not invade agar, and did not form biofilms, while the susceptible strains exhibited the opposite behaviour. Through microfluidics experiments, we observed that both the susceptible and FNS strains were capable of adhering to a plastic surface under dynamic conditions, but the FNS strains formed unstable aggregates that did not remain attached to the surface, confirming the filamentation defect of these strains. In the second part of the study, we observed that FNS strains are susceptible to clinical disinfectants, although they presented a slight resistance to some of them, such as chlorhexidine, compared to susceptible isolates. In this work, we address important aspects to understand the dissemination of FNS strains in clinical outbreaks.

## 1. Introduction

*Candida parapsilosis* is a pathogenic yeast responsible for invasive diseases, primarily affecting immunosuppressed individuals. Its prevalence is strongly linked to the use of medical devices, such as parenteral nutrition systems and central venous catheters (CVCs), due to its capacity to form biofilms on these surfaces [1,2,3]. Furthermore, *Candida parapsilosis* can readily spread within hospital settings, leading to outbreaks. Evidence suggests that horizontal transmission often occurs, with healthcare workers’ hands playing a significant role in its dissemination [4]. Worldwide, the incidence of *C. parapsilosis* is variable, but it ranges between the first to the third cause of candidemia, depending on the geographical region [5,6,7,8,9]. However, its incidence increases among neonates, being one of the main concerns among pathogenic fungi in this type of patient [10].

Similarly to *C. albicans*, *C. parapsilosis* is a diploid yeast that belongs to the CTG clade. During stress conditions, it can develop pseudohyphae [5,11,12]. One of the interesting features of this yeast is its reduced in vitro susceptibility to echinocandins (caspofungin, micafungin and anidulafungin).

Although this species is considered susceptible to other antifungal families, mainly azoles and polyenes, in recent years there has been increasing worldwide concern about the global emergence of resistance to azole antifungal drugs, in particular to fluconazole [8,13,14,15,16,17,18,19,20]. In many cases, these resistant isolates cause clinical outbreaks that are difficult to manage [14,21,22,23,24,25,26,27,28]. A worrisome situation has been detected in Spain since 2020, concurrently with the impact of the COVID-19 pandemic, where several outbreaks in different geographical areas (including Madrid, the Balearic Islands, Cataluña, Castilla y León and Cantabria) have been reported [29]. Interestingly, there was an association between specific genetic clones and dispersion among hospitals of the same city and geographical region [21,25,29,30].

Azole antifungals inhibit the enzyme 14-sterol-α-demethylase (encoded in yeasts by the *ERG11* gene), which is required for ergosterol synthesis. Resistance to azoles can be caused by multiple mechanisms. Some of them involve mutations in the target enzyme, with Y132F, K143R and G458S being some of the most common mutations [16,17,19,22,31,32,33,34]. Other mechanisms involve mutations that affect the expression of membrane efflux pumps (mainly ABC and MDR transporters) that expel the antifungal to the extracellular medium [35,36,37]. Some of the main mutations in this case have been described in transcription factors that regulate the expression of these pumps, such as Tac1, Mrr1 and Upc2 [34,35,38,39].

The emergence of these outbreaks raises several challenges with important clinical implications. The increase in FNS isolates could potentially limit the antifungal treatment options for these patients, but it is not known which of the remaining therapies (mainly echinocandins or the liposomal formulation of amphotericin B) are the most adequate to treat these patients. Furthermore, it is necessary to investigate which are the best clinical measures to control and eradicate the possible sources of infection in the hospital environment. But another interesting unsolved question is to determine why these clinical outbreaks are being caused by clonal isolates with reduced susceptibility to azoles more frequently than susceptible isolates. Different studies indicate that selection of resistant strains was not due to an increase in antifungal use in affected centers or to previous administration of azoles to the patients [29,40]. In contrast, it has been hypothesized that the situation found in Spain was a consequence of the clinical impact of the COVID-19 pandemic [29]. This is supported by the fact that *C. parapsilosis* infections are associated with the use of medical catheters and intubation. But still, there is an intriguing question, which is why in these conditions there has been an emergence of the FNS strains and not of the wild-type susceptible isolates.

In this work, we used a collection of FNS and susceptible *C. parapsilosis* strains, received at the Spanish Mycology Reference Laboratory (SMRL) during 2020–2022 from various Spanish hospitals. We investigated some of the traits that are involved in virulence (growth, filamentation, biofilm formation, invasion and adhesion) in a collection of *C. parapsilosis* FNS strains and susceptible strains to determine if FNS isolates present a different phenotype that correlates with the appearance of outbreaks. We also analysed the resistance of both types of strains to some of the disinfectants used in clinical settings (ethanol, hydrogen peroxide, chlorhexidine, quaternary ammonium and sodium hypochlorite) to determine the cleaning products that could be potentially useful to eradicate FNS isolates from the hospital environment.

## 2. Materials and Methods

### 2.1. Strains and Growth Conditions

A list of the *C. parapsilosis* strains can be found in Supplemental Table S1. All the *C. parapsilosis* isolates were characterized by sequencing of the ITS region of the ribosomal DNA [41] and identified as *C. parapsilosis* sensu stricto We included 83 FNS isolates (belonging to different genotypes) and 59 susceptible strains. The FNS strains harboured mainly the Erg11^Y132F^ mutation (65 isolates, representing genotypes 10, 67 and 96), but we also included 12 with the Erg11^G458S^ substitution (genotype 54), one with K143R mutation and 5 FNS strains without mutation in *ERG11* gene (wild type, WT-R). Regarding the origin of the strains, we included invasive and superficial samples, and also strains from environmental sources (Supplemental Table S1). Description of the genotypes and their geographical association with Spanish hospitals is described in [29]. The yeasts were routinely maintained in liquid Sabouraud medium (Oxoid) at 30 °C with moderate shaking (150 r.p.m). Additionally, solid Sabouraud containing 1.5% agar was also used.

### 2.2. Antifungal Susceptibility Testing in Microdilution Plates (EUCAST Protocol)

Antifungal susceptibility testing was performed according to EUCAST protocol [42]. The medium (RPMI 1640 medium (Merck, Sigma-Aldrich, Saint Louis, MI, USA) was buffered with MOPS (Merck, Sigma-Aldrich) at pH 7 and supplemented with 2% glucose (Merck, Sigma-Aldrich). The antifungals used and concentration ranges were as follows: (AmB, Merck, Sigma-Aldrich, 16–0.03 mg/L), flucytosine (64–0.125 mg/L), fluconazole (FLC, Merck, Sigma-Aldrich, 64–0.125 mg/L), itraconazole (ITZ, Janssen Pharmaceutical Research and Development, Beerse, Belgium, 8–0.016 mg/L), voriconazole (VOR, Pfizer Pharmaceutical Group, New York, USA, 8–0.016 mg/L), posaconazole (POS, Merck, Sigma-Aldrich, 8–0.016 mg/L), isavuconazole (ISV, Pfizer Pharmaceutical Group, New York, USA, 8–0.016 mg/L), caspofungin (CSP, Merck, Sigma-Aldrich, 16–0.016 mg/L), micafungin (MICA, Astellas Pharma Inc., Tokyo, Japan, 2–0.004 mg/L) and anidulafungin (ANID, Pfizer Pharmaceutical Group, New York, USA, 4–0.008 mg/L). The Minimal inhibitory concentration (MIC) was estimated as the concentration that caused 50% of growth inhibition compared to the control well without antifungal, except for amphotericin B (90%). Strains were categorized as susceptible (S), resistant (R) or susceptible increased exposure (I) as described by the breakpoints from EUCAST (see https://www.eucast.org/astoffungi/clinicalbreakpointsforantifungals, accessed on 10 June 2023). In all the antifungal susceptibility assays we included the *C. parapsilosis* ATCC 22019 and *C. krusei* ATCC 6258 strains as quality controls to verify the proper preparation of the plates and of the results obtained in each plate.

### 2.3. Growth Curves

The yeast strains were incubated in liquid Sabouraud as described above overnight, and then they were washed and suspended in sterile distilled water at 2 × 10^5^ cells/mL. A total of 100 µL from this inoculum was added to 100 µL of RPMI medium 1640 (Merck, Sigma-Aldrich) buffered with MOPS (Merck, Sigma-Aldrich) at pH 7, and supplemented with 2% glucose (Merck, Sigma-Aldrich) into 96-well plates (Falcon, Tamaulipas, Mexico). The plates were covered with a gas-permeable membrane (Breathe-easy sealing membrane, Merck, USA) to avoid evaporation and contamination during the incubation, and then was placed into a Multiskan FC spectrophotometer (Thermo Fisher Scientific, Warrington, UK) where it was incubated at 35 °C. Optical density (O.D.) at 540 nm was measured every hour for 48 h. The data were exported and analysed with GraphPad Prism 9.0 (GraphPad Software, Boston, MA, USA. The Gompertz method (Y = YM × (Y0/YM)^(exp(-K × X))) was applied to estimate the parameters of the growth curve, and the value of the Ymax (maximal absorbance of the culture) was represented (see https://www.graphpad.com/guides/prism/9/curve-fitting/reg_gompertz-growth.htm for further details, accessed on 15 November 2024).

### 2.4. Real Time Microscopy

Yeast cells were incubated overnight in liquid Sabouraud at 30 °C as described above, and then washed and suspended in RPMI medium (Sigma Aldrich-Merck) containing 2% glucose buffered at pH 7 with 80 mM MOPS buffer at 10^5^ cells per mL. A total of 200 mL from this suspension was placed in 96-well plates (Falcon TC treated plates, plastic, flat-bottom) and incubated at 35 °C in the chamber of a Thunder microscope (Leica microsystems, Wetzlar, Germany) with humidity. Images were taken every 10 min for 15 h. We included the ATCC 22019 strain in all the experiments as a control for pseudohyphae forming. Videos were processed using FIJI software v1.53c (https://fiji.sc, accessed on 20 January 2021).

### 2.5. Biofilm Formation and Measurement with XTT

Biofilm formation was assessed following the protocol described in [43]. Yeast cells were incubated in Sabouraud and inoculated in RPMI as described above in 96-well plates in triplicates. The plate was incubated at 37 °C for 48 h. After this incubation time, we measured the absorbance at 540 nm in the Multiskan FC (Thermo Fisher Scientific) to obtain an estimation of the total biomass (including planktonic cells and biofilm) of the well. Then, the wells were washed vigorously three times with 200 µL of PBS (Phosphate Buffered Saline). Finally, 100 µL of PBS and 50 µL of Cell Proliferation kit II (XTT) (Roche Mannheim, Germany) were added to each well. As controls, the same amount of PBS and reagent were added to empty wells. After 2 h of incubation at 37 °C, the absorbance was measured at 450 nm in the Multiskan FC (Thermo Fisher Scientific).

To quantify the amount of biofilm formed, the mean absorbance of the negative control was subtracted from each well. Then, the absorbance at 450 nm was divided by the absorbance at 540 nm obtained before washing the wells to normalize the amount of biofilm by the total mass of the well. Finally, the mean and standard deviation were calculated and plotted using GraphPad (GraphPad Software, Boston, MA, USA. Strains were arbitrarily categorized as high-biofilm-forming (normalized O.D. > 0.5), low-biofilm-forming (normalized O.D. between 0.15–0.5) and non-biofilm-forming (O.D. < 0.15).

### 2.6. Agar Invasiveness

The ability to invade agar solid medium was evaluated as described in [44,45] with some modification. The strains were grown in Sabouraud overnight and suspended in PBS at 10^5^ cell/mL as described above. Then, 10 µL were placed onto agar Sabouraud medium. The plates were incubated at 30 °C for 48 h. After this time, we took a photo with a Nikon camera with a macro lens. Then, the agar surface was gently washed five times with PBS, and, finally, another photo was taken to visualize the degree of yeast invasion into the agar. We included the ATCC 22019 strain in all the experiments as control for high invasion. Strains were arbitrarily categorized as high-invasive, low-invasive and non-invasive depending on the amount of yeast visualized in the agar.

### 2.7. Microfluidic Experiments

Cells incubated in Sabouraud as described above were suspended in RPMI medium at 10^5^ cell/mL and placed in a 15 mL Falcon tube. To perform the microscopy, we used 6-well channel μ-Slides (Ibidi, Gräfelfing, Germany). In these slides (glass bottom), the channel, where visualization occurs, is connected at each side with wells, so a medium can be injected through one side and discarded through the other opposite well. One of the side wells was connected with a Falcon tube containing the medium (with or without cells) using 0.8 mm diameter silicon tubing, and the other side-well was connected to a 20 mL syringe using the same tubing. The syringe was placed in a Legato 210 pump (KD Scientific), which caused aspiration of the medium at a 20 µL/min flow rate. The slide was placed in a Thunder Microscope (Leica Microsystems) at 35 °C, and the system was first purged with fresh RPMI medium for 30 min. Then, one of the tubes was included in the 15 mL Falcon tube containing the cells. Acquisition was performed over three hours in bright field taking images every 10 min. Then, the tubing was transferred to a 50 mL Falcon tube containing fresh medium without cells, and acquisition continued for 15 more hours taking images every 10 min. The images were exported and videos created using FIJI software v 1.53c. In all the experiments, we included the CL-11485 and CL-11366 strains as controls for adherent cells and big aggregates, respectively.

### 2.8. Susceptibility to Disinfectants

The strains were grown on Sabouraud agar plates for 24 h at 30 °C. Susceptibility to clinical disinfectants was evaluated as described in [46] with minor modifications. An inoculum of 10^6^ cells/mL was prepared in the selected disinfectants at various concentrations. These included ethanol (Merck) at 50%, hydrogen peroxide (Merck Sigma Aldrich) at 15%, sodium hypochlorite (Merck Sigma Aldrich) at 25%, chlorhexidine digluconate (Merck Sigma Aldrich) at 10%, and two different compounds containing quaternary ammonium: Zwittergent 3–14 detergent (Merck Sigma Aldrich) at 1% and Surfanios Premium (Anios Laboratories, Lille, France) at 0.008%. After preparing the inoculum, the cells were incubated with the disinfectant for a specific duration: ethanol, sodium hypochlorite, hydrogen peroxide and Surfanios for 1 min; chlorhexidine for 2 min; and Zwittergent for 1 h. All the incubations were performed at room temperature. After the indicated times, a 1/200 dilution was rapidly performed in water to dilute the disinfectants, and 100 µL was plated on Sabouraud agar plates. In every experiment, similar samples were carried out in water without any disinfectant as viability controls. The plates were incubated at 30 °C for 48 h and the number of colony-forming units (CFUs) was determined. Viability in the presence of each disinfectant was expressed as the percentage of colonies obtained compared to the number of colonies in the control plates.

### 2.9. Statistical Analysis

We first assessed the normal distribution of the samples using the Shapiro–Wilk test. For those cases in which there was a normal distribution, we used the Student *t*-test to compare the samples. For those cases in which the samples were non-normally distributed, we used the Mann–Whitney non-parametric test. All the statistics were performed using GraphPad 9.0 software (GraphPad Software, Boston, MA, USA).

## 3. Results

We investigated different phenotypic traits of FNS strains that could have an impact on their clinical behaviour (including antifungal susceptibility, biofilm, morphology, agar invasiveness and susceptibility to disinfectant). For this purpose, we included a set of susceptible strains (*n* = 59) and FNS isolates (*n* = 83) from different genetic clones and harbouring different mutations in the *ERG11* gene, causing mainly the Erg11^Y132F^ (*n* = 65, including both heterozygous and homozygous) and Erg11^G458S^ (*n* = 12). Additionally, we included one FNS strain with the Erg11^K143R^ mutation and five with no mutation in the *ERG11* gene (WT-R).

### 3.1. Antifungal Susceptibility of FNS C. parapsilosis Isolates

We first characterized the susceptibility of all the isolates to different antifungals (see Supplemental Table S1). We paid particular attention to whether the mutations in the Erg11 protein (Y132F, G458S and K143R) had any influence on the susceptibility to Amphotericin B and echinocandins. We specially investigated the susceptibility to Amphotericin B, since this antifungal binds to ergosterol, whose synthesis is inhibited by azoles. As shown in Supplemental Table S1 and summarized in Table 1, fluconazole susceptible and FNS strains were susceptible to Amphotericin B, independently of the mutation at the *ERG11* gene. Similarly, the susceptibility to other antifungals (flucytosine or echinocandins) was not affected by mutations in the *ERG11* gene (Table 1 and Supplemental Table S1).

### 3.2. Characterization of Growth of Susceptible and FNS Strains 

**Figure 1:**
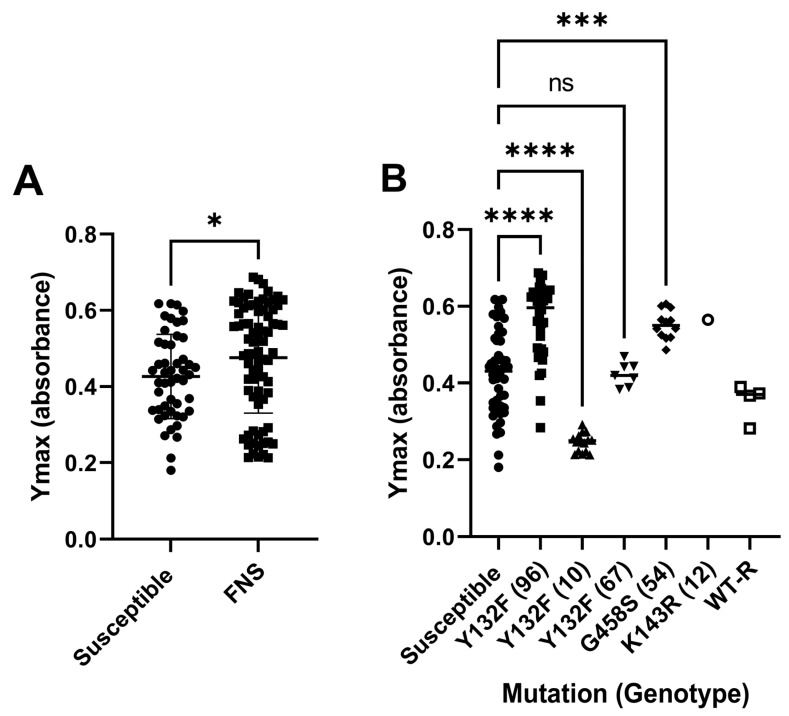
Growth of susceptible and FNS strains and biofilm formation. (**A**) Cells were incubated in RPMI medium with 2% glucose at pH 7 as described in Section 2 and incubated at 35 °C in a Multiskan FC spectrophometer, where growth was monitored every hour at 540 nm. (**B**) Data from the A, but separated by resistance mutations and genotypes. The graphs show that the value of the calculated Ymax was calculated after adjusting the growth curves to the Gompertz model. Statistical differences, determined by t-test or ANOVA, are denoted by asterisks as follows: *p* ≤ 0.05 (*), *p* ≤ 0.001 (***), *p* ≤ 0.0001 (****); ns = not significant (*p* > 0.05).

We next performed growth curves in liquid medium (RPMI medium) to investigate if there was a difference in the duplication time of the susceptible and FNS isolates (see M&M). We represented the Ymax value calculated from each curve, which is an estimation of the final amount of biomass and OD in each well. As shown in Figure 1A, the FNS had a higher Ymax than the susceptible strains, (Figure 1A). However, we noticed that there was a great variation in the values obtained in the resistant strains, so we analysed the data depending on the resistant mutations and genotypes used. As shown in Figure 1B, we found that the growth was dependant on the genotype. Genotype 96 containing the Y132F mutation (*n* = 38) and 54 harbouring the G458S mutations (*n* = 12) reached a higher absorbance in the growth curves. In contrast, strains from the genotype 10 (harbouring the Y132F mutation, *n* = 15) significantly had a lower Ymax, suggesting that they had a growth defect. No differences were found in isolates from genotype 67 (*n* = 6) compared to the susceptible strains.

### 3.3. Pseudohyphae Formation

*Candida parapsilosis* can form pseudohyphae in different media, such as RPMI, which are considered important for virulence since they promote adhesion and invasion. For this reason, we first investigated the morphology of susceptible and FNS strains using real-time microscopy. After examination of the videos of all the isolates, we found that there was a variety of morphologies among isolates. In general, we found two phenotypes: strains with the ability to filament (either with long or short pseudohyphae) and isolates that remained in the yeast form during the whole incubation and did not induce any morphological change (Supplemental Video S1). When we correlated the morphology with the susceptibility profile of the strain, we found that around that 97% of the FNS isolates did not form any type of filamentation and were always observed as budding yeasts. Considering the mutation in the Erg11 protein, all strains (Y132F, G458S, K143R, and three WT-R) remained in yeast form, except for two WT-R strains that filamented. In contrast, for susceptible strains, around 75% of the strains were able to develop pseudohyphae (Table 2). This result suggested a strong association between a defect in filamentation and resistance to fluconazole.

### 3.4. Biofilm Formation

**Figure 2:**
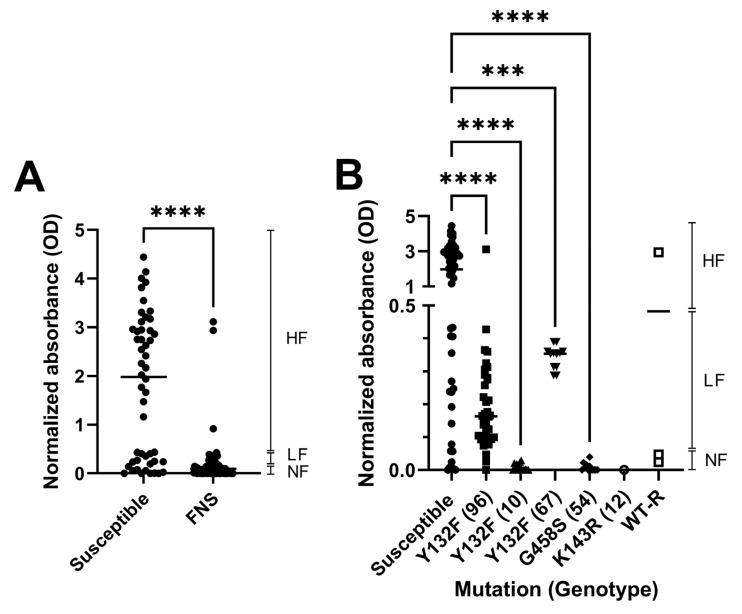
Biofilm formation by susceptible and FNS *C. parapsilosis* strains. (**A**) Biofilm was measured as described in Section 2 in susceptible and FMS strains. The normalized XTT absorbance is shown (XTT reduction/O.D. of the well before washing), and thresholds for high-biofilm-forming (normalized O.D. > 0.5), low-biofilm-forming (normalized O.D. between 0.15 and 0.5) and non-biofilm-forming (O.D. < 0.15) were established. (**B**) Data from A were separated according to the resistance mechanisms and genotypes. Statistical differences, determined by t-test or ANOVA, are denoted by asterisks as follows: *p* ≤ 0.001 (***), *p* ≤ 0.0001 (****).

We next investigated the ability of the *C. parapsilosis* strains to form biofilms in 96-well plates. The cells were prepared and suspended in RPMI as described above, and incubated at 35 °C for 48 h without shaking and then washed extensively to remove planktonic cells. The viability of the remaining biofilm was quantified using XTT. The normalized XTT absorbance is shown (XTT reduction/O.D. of the well before washing) in Figure 2. We classified the strains based on the amount of biofilm formed as follows: high-biofilm-forming (normalized O.D. > 0.5), low-biofilm-forming (normalized O.D. between 0.15 and 0.5), and non-biofilm-forming (normalized O.D. < 0.15). As shown in Figure 2A, the amount of biofilm observed in susceptible isolates was variable, although around 75% of these isolates were categorized as high-biofilm-forming strains. In contrast, the great majority of FNS isolates did not form biofilms, and only a small percentage were categorized as low-biofilm-forming strains (Table 2). Considering the Erg11 mutations, all G458S, K143R, two WT-R and Y132F strains from genotypes 10 were non-biofilm-forming, while Y132F strains from genotypes 96 and 67 were low-forming. Interestingly, three WT-R strains were high-forming.

### 3.5. Agar Invasiveness

**Figure 3:**
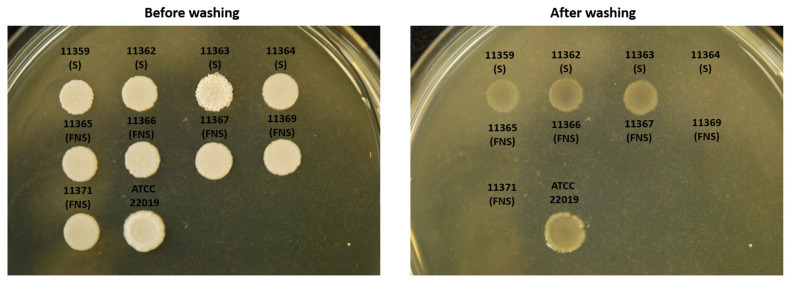
Visualization of ability of *C. parapsilosis* strains to invade agar plates (see M&M). (**Left image**), plate before washing, (**right image**), plate after extensively washing the plate.

The invasiveness capacity of FNS strains was investigated as adhesion and filamentation are traits associated with tissue invasion during infection (Figure 3). We observed that there was a variation in the ability to invade the agar among *C. parapsilosis* isolates. For susceptible isolates, around 75% of the isolates were able to invade the agar, whereas around 93% of FNS strains could not invade it, which correlated with the lack of filamentation and biofilm formation exhibited by these strains (Figure 3 and Table 2). With the exception of three WT-R strains and three isolates with the Y132F mutation, none of the remaining FNS strains were able to invade the agar (Figure 2).

### 3.6. Adhesion Under Dynamic Conditions

**Figure 4:**
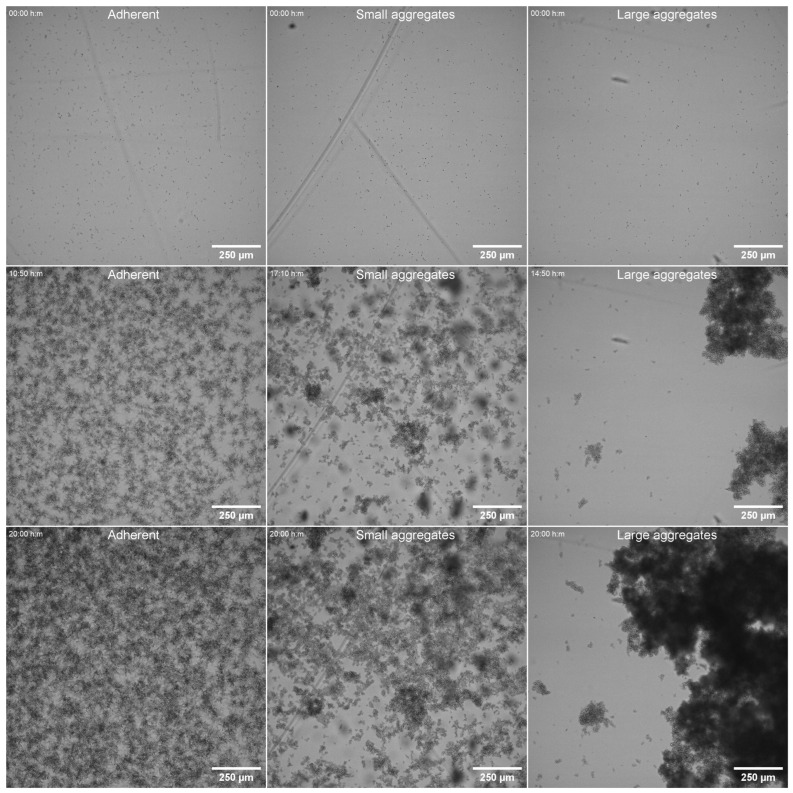
Visualization of different strains under dynamic conditions over time. Different *C. parapsilosis* strains showing different phenotypes were adhered to a plastic surface as described in M&M, and then a constant flux of medium was applied overnight. The images show the phenotypes observed over time. (**Left images**) cells strongly adhering to the surface that develop pseudohyphae, center and right, two different strains that do not form pseudohyphae, and form small (**center images**) or large aggregates (**right images**) that move with the flux. The different images in the same column represent different times of the same strain and well position.

To further characterize the ability of FNS isolates to adhere to and colonize the solid environment, we investigated the binding of *C. parapsilosis* cells by real-time microscopy in dynamic conditions, where an efflux pump was connected to well-chambers and caused the movement of the media during acquisition in the microscope. We conducted this experiment with 22 strains: six susceptible and 16 FNS strains. Among the FNS strains, we analysed four G458S, the K143R isolate, and 11 Y132F strains from the three main genotypes (10, 67 and 96). Surprisingly, we found that FNS isolates were able to bind the surface similarly to susceptible strains in short times in the yeast form. In our experimental approach, after initial binding, we removed the yeast suspension of the circuit and continued the acquisition with a fresh medium also under dynamic conditions, so we could observe the behaviour of the cells that had adhered to the plastic surface. In this way, we found that susceptible strains started developing pseudohyphae after a few hours and most of the cells grew attached to the slide. In the case of FNS strains, none of the strains tested were able to filament, and during the incubation they grew as clumps of variable size of yeasts that formed unstable biofilms that eventually detached after approximately 10–15 h of incubation (Figure 4 and Supplemental Video S2). However, among FNS strains, we observed two different types of clumps and aggregates. The isolates harbouring the G458S (genotype 54) and Y132F from genotypes 10 and 67 formed very large clumps, which ranged between 500 and 1000 µm. In contrast, the clumps observed in the strains with the Y132F mutation from genotype 96 were smaller (between 30 and 100 µm, Figure 4).

### 3.7. Susceptibility of C. parapsilosis to Clinical Disinfectants

In the clinical context, a relevant issue in controlling the development of *C. parapsilosis* outbreaks is to establish the best conditions to eliminate the environmental sources of infection using appropriate disinfectants. We wanted to analyse whether FNS strains were susceptible to some of the most common clinical disinfectants used in clinical practice, to determine if for these particular strains, specific measures must be considered. Due to technical limitations, we focused on a set of resistant strains harbouring the Y132F mutations from the three major genotypes because they are the most widely distributed in Spain. We investigated the susceptibility to ethanol, sodium hypochlorite, chlorhexidine, hydrogen peroxide and quaternary ammonium compounds.

**Figure 5:**
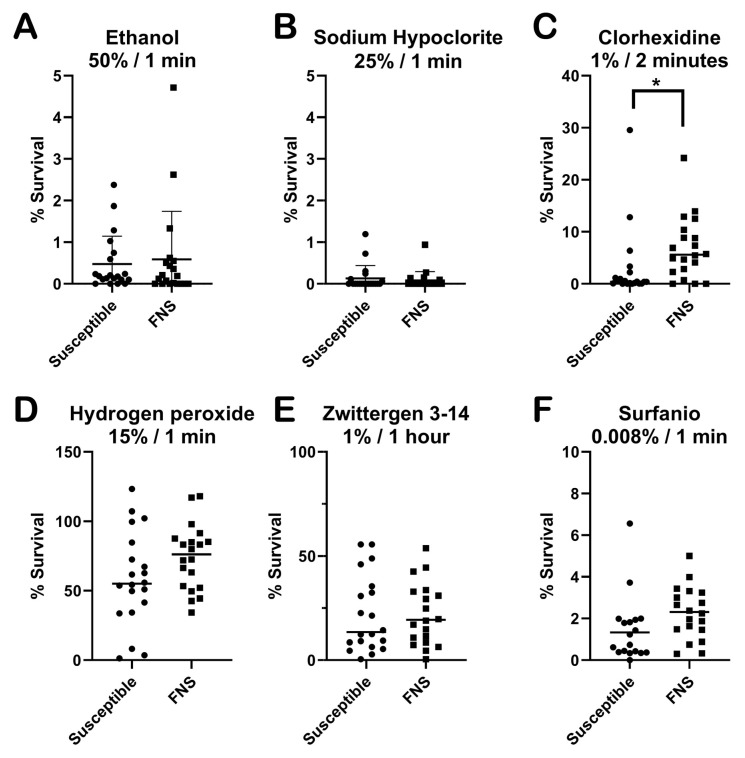
Killing assays of susceptible (*n* = 20) and FNS (*n* = 20) *C. parapsilosis* strains in the presence of different clinical disinfectants. The graphs show the survival percentage of each strain analysed. Statistical differences were assessed using the Mann–Whitney non-parametric test. All the incubations were performed at room temperature for the time indicated in each case. *, *p* < 0.05. (**A**) Susceptibility to Ethanol; (**B**) susceptibility to sodium hypochlorite; (**C**) susceptibility to Clorhexidine; (**D**), susceptibility to Hydrogen peroxide; (**E**) susceptibility to Zwittergen 3-14; (**F**) susceptibility to Surfanios. Concentrations of each disinfectant and incubation times are stated above each graph and in Section 2.

As shown in Figure 5, ethanol and sodium hypochlorite presented a strong fungicidal effect against both susceptible and FNS strains. In the case of chlorhexidine, all the strains showed a low survival rate against this compound, but FNS showed a slightly higher survival than susceptible isolates (*p*-value, 0.0073). A similar situation was found in the presence of hydrogen peroxide and quaternary ammonium. For these two disinfectants, the survival of all the isolates was higher compared to the other substances, and a trend of higher survival was also observed in FNS strains, although due to the larger variation there was no statistical difference between the groups. We also tested another reagent which contained several disinfectants, including quaternary ammonium, which showed a potent fungicidal activity. For this last case, survival of the strains was only observed in the presence of very low concentrations (below 0.01%), and again, a non-significant trend of higher survival was found among the FNS isolates.

## 4. Discussion

The epidemiology of fungal diseases has undergone several changes in recent decades. Among them, the increase in the incidence of *Nakaseomyces glabratus* (formerly *C. glabrata*) [6], the emergence of azole resistance in *Aspergillus fumigatus* [47,48] and the appearance of *Candidozyma auris* (formerly *Candida auris*) as a human pathogen [49] are some of the threats to the immunocompromised. In general, these changes have in common the selection of resistance to antifungals, with the most frequent selection being the resistance to azoles and, in particular, fluconazole. In this sense, in the last few years, there has been a dramatic increase in reports describing the selection of fluconazole-resistant *C. parapsilosis* [13]. Furthermore, this selection is commonly associated with difficult-to-control clinical outbreaks. This highlights a new challenge in the field of medical mycology, which warrants detailed studies to investigate this phenomenon. Interestingly, a high resistance rate to fluconazole has recently been reported among environmental *C. parapsilosis* strains isolated from niches such as faeces of synanthropic birds and fruits [50,51], which raises further concern about the global spread of fluconazole adaptation.

The reasons for the selection of FNS *C. parapsilosis* isolates are unknown. A previous report indicated that the expansion of resistant strains is not associated with selection by antifungal pressure [29,40]. This suggests that FNS isolates may possess certain phenotypic traits that facilitate their dissemination in the hospital environment. For this reason, in this work we investigated if these FNS strains had a difference in some phenotypic characteristics that may favour their dispersion in the hospital environment, including filamentation ability, biofilm formation in both static and dynamic conditions and resistance to clinical disinfectants.

We found that there were some differences in the in vitro growth of the FNS isolates defined as the Ymax (final O.D). For example, two FNS genotypes (96 and 67) showed a slightly better growth than susceptible strains. In contrast, FNS strains from genotype 10 exhibited a decreased growth. In consequence, our data indicate that expansion of FNS in the hospital environment is not correlated with the growth of the isolates, but further studies are needed to investigate whether these parameters are related to the fitness or virulence of the strains.

Pseudohyphae formation is an important phenotypic feature found in *C. parapsilosis,* which is involved in adhesion and invasion. A recent study describes that the ability of *C. parapsilosis* to induce this transition depends on the medium, being more prominent in low-nutrient media, such RPMI with 0.2% glucose [12]. In our case, we used RPMI medium supplemented with 2% glucose, and found that in this medium this species can also form pseudohyphae. Despite variations in the behaviour of the different susceptible strains, in general, most of them (around 75% of the tested isolates) were able to form pseudohyphae and biofilm under static conditions. Strikingly, the vast majority of the FNS isolates showed a very similar behaviour pattern. They did not form pseudohyphae, nor did they have a significant ability to form biofilms after 48 h of incubation in plastic plates. Interestingly, the lack of biofilm was not correlated to the ability to adhere to surfaces of the yeast forms. This was demonstrated in the microfluidic experiments, where we observed that all the strains, regardless of their ability to form pseudohyphae or their resistant phenotype, were able to bind to the surfaces under dynamic conditions. However, the strains that did filament produced aggregates of yeast forms that became unstable and prone to detach from the surface. Although these results were initially unexpected, they suggest a hypothesis in which the individual cells could adhere to surfaces as most of the susceptible isolates, but then could disseminate through the hospital environment more easily. We believe that a similar situation could also apply to medical implants, such as catheters.

Previous studies have also found that strains harbouring the Y132F mutation exhibit a reduced ability to form biofilm compared to other strains with different resistant mechanisms [22] or to susceptible isolates [52]. In agreement, a study has described the presence to two *C. parapsilosis* phenotypes (floating and sinking), which depends on the ability of the strains to float or rapidly sediment in CLSI plates. These authors described that most of the FNS strains presented the sinking phenotype, which was associated with less pseudohyphae formation and reduced virulence in *G. mellonella* [53]. However, other studies did not find a correlation between fluconazole resistance and reduced ability to form biofilms compared to susceptible strains [40,54], which was even higher in the presence of fluconazole. A recent study also reported that *C. parapsilosis* strains that present several resistance mechanisms to azoles or echinocandins do not have a reduced ability to form biofilms, but show increased resistance to the antimicrobial effects of neutrophils [55]. All these data suggest that the genetic background of the isolates is a key determinant in the virulence phenotype of the FNS strains. In our study, we included FNS strains belonging to different genotypes and also with three different resistance mechanisms, observing a similar behaviour in all of them. At the moment, there are two possible explanations to understand the association between the defects in biofilm and filamentation and resistance. One possibility is that mutations in the Erg11 protein cause an imbalance in the ergosterol metabolism that affects the ability to induce morphological transitions. The relationship between the presence of Erg11 mutations and sterol metabolism is very scarce. A study with the related species *C. tropicalis* showed that in a set of resistant strains to fluconazole there was an increase in respiration rate and ergosterol content [56]. Recently, it has been shown that isolates with the Y132F/R398I mutations present some differences in lipid metabolism compared to a WT strain [54]. In our case, we have observed that FNS strains also have a slight defect in the growth rate compared to the susceptible isolates, consistent with the idea that these strains present other defects related to ergosterol metabolism which might influence the fitness of these strains. But another possibility is that FNS strains are derived from clones which originally have other defects. In agreement with this idea, we found that around 25% of susceptible isolates were also defective in filamentation and biofilm formation, which is a significant percentage of strains. For this reason, we argue that the capacity for biofilm formation is highly variable among susceptible isolates, and might reflect that a significant proportion of strains are more likely to accumulate mutations that have multiple and pleiotropic effects. For these reasons, future studies are warranted to investigate in detail the genetic determinants associated with the resistant phenotypes. For example, the generation of filamentation mutants in different genetic backgrounds using molecular biology techniques and investigation of the acquisition of fluconazole resistance might provide new information about the associations described in this article.

Another aspect that is critical for understanding the possible adaptation of FNS isolates is their susceptibility to clinical disinfectants. In this study, we have investigated this issue with a subset of susceptible and FNS strains. In general, the most effective disinfectants against *C. parapsilosis* were ethanol and sodium hypochlorite (common bleach). Regarding the other disinfectants, our data suggest that FNS strains show a slightly higher survival against some clinical disinfectants (in particular chlorhexidine, hydrogen peroxide and quaternary ammonium) when compared to susceptible strains. For these reasons, our data support that in the context of a clinical outbreak caused by FNS *C. parapsilosis* isolates, the use of these disinfectants should be reviewed to ensure the elimination of the fungal cells (i.e., repeated cleaning of surfaces). The fact that FNS strains showed a trend toward higher survival against some disinfectants, such as hydrogen peroxide, also opens a new research field to investigate whether they are also more difficult to eliminate by the antifungal mechanisms induced by our immune system. This topic should be the primary focus of future research.

In conclusion, our data show that the accumulation of mutations conferring resistance to fluconazole may have pleiotropic effects that influence the ability of *C. parapsilosis* strains to induce phenotypes related to virulence and adaptation to the clinical environment. This work opens new research topics to fully investigate the genetic determinants that correlate resistance and virulence in *C. parapsilosis*, and to define future strategies to reduce the impact of these strains in clinic.

## Figures and Tables

**Table 1:** Effect of fluconazole resistance and Erg11 mutation on susceptibility to Amphotericin B. nd, not determined.

	Susceptible (*n*)	FNS Non Susceptible (*n*)				
AmB MIC	WT	Y132F (Heterozygous)	Y132F	G458S	K143R	WT
0.06	2					
0.125	8		2	1		
0.25	30	2	18	6		2
0.5	14	10	23	5		1
1	5		6		1	2
Total	59	12	49	12	1	5
Min-Max	0.06–1	0.25–0.5	0.125–1	0.125–0.5	1–1	0.25–1
Mode	0.25	0.5	0.5	0.25	nd	nd

**Table 2:** Percentages of strains that induced morphological transitions (pseudohyphae vs yeast), formed biofilm (high-forming, low-forming or non-forming) and that invaded or did not invade the agar among susceptible or fluconazole-non-susceptible (FNS) strains.

		% of Strains	
		Susceptible (*n* = 59)	FNS (*n* = 79)
Morphology	Pseudohyphae	73	3
	Yeast	27	97
Biofilm	High-forming	63	2
	Low-forming	21	31
	Non-forming	16	67
Agar invasiveness	Invasion	97	31
	No invasion	3	69

## Data Availability

Dataset available on request from the authors.

## Supplementary Materials

The following supporting information can be downloaded at: https://www.mdpi.com/article/10.3390/jof11040299/s1. Table S1: summary of all the strains, genotypes and phenotypes analysed in this work; Video S1: visualization of different behaviours (pseudohyphae and yeast) formed by *C. parapsilosis* in RPMI medium at 35 °C; Video S2: visualization of the behaviour of different strains in microfluidic conditions.
